# Women in science: myth, harsh reality, or advantage

**DOI:** 10.3389/fnhum.2023.1247242

**Published:** 2023-10-25

**Authors:** Ruth Feldman

**Affiliations:** ^1^Center for Developmental Social Neuroscience, Reichman University, Herzliya, Israel; ^2^Child Study Center, Yale University, New Haven, CT, United States

**Keywords:** women, history of science, phenomenology, gender equality, biobehavioral synchrony, affiliative neuroscience, oxytocin, parental brain

## Abstract

To initiate discussion on women in science, we begin with Gerald Edelman’s definition: “Science is imagination in the service of the verifiable truth,” which underscores “verifiability,” truth reached by evidence, as the pathway science charts to Truth. “Verifiability” is named after the Roman Goddess Veritas, the daughter of Cronos and the mother of Virtus, suggesting that mythology viewed science as embodied by a female, embedded in its historical time, and aimed to breed values. We contemplate three perspectives on the topic and discuss their potential risks. The *Veracity (Veritas) Perspective* holds that science is impartial to the gender, race, political camp, or religious affiliation of its practitioner and from this perspective “women in sciences” is an oxymoron; science is, essentially, genderless. We argue that this perspective is misleading. Becoming a scientist requires education, resources, encouragement, training, role models, time, and funding, and the lack of such provisions banned women from the gates of Truth. The *Harsh Reality* perspective brings data presenting a grim picture. From 1902 to 2022 only 3.6% of Nobel Prizes in sciences were awarded to women and percentages of women in top academic positions are a third or lower across the US and Europe despite earning about 50% of PhDs in sciences. We contemplate internal and external reasons for this reality. Finally, the *Potential Advantage* position asks whether women may have unique sensitivities in the road to cumulative knowledge. We base our discussion on 20th century philosophical models that call to move from the metaphysical and abstract to the daily and contextual in the acquisition of knowledge and on research describing the distinct neural pathways to motherhood and fatherhood. We conclude by highlighting our unique historical time and the emergence of novel topics in neuroscience through the work of female and male scientists; interaction synchrony, inter-brain communication, and social and affiliative neuroscience.

To my four daughters

Esti, Tamar, Naama, and Yael

Women with a room of their own

## Introduction

Perhaps the best entry-point to the topic “women in science” is finding a good definition to the question: “what is science.” For me, the most poignant definition is the one Gerald Edelman delivered in his Nobel Prize acceptance speech (Edelman, 1972); “science is imagination in the service of the verifiable truth.” Apart from its sheer elegance and the perfect use of Popper’s (1959) “parsimony principle,” the definition contains two core components that pinpoint Edelman’s wise perspective on science. First, he suggests that if our journey begins with imagination and its destination is Truth, science charts only one pathway among several others, indicating that science does not have sovereignty on truth. Second, the path charted by science is marked by “verifiability.” Such “verifiable truth,” truth achieved through evidence, differentiates science from other forms of truth, such as existential truth, artistic truth, religious truth, or heuristic truth. “Verifiability,” then, becomes the cornerstone of the human scientific project.

Verifiability, the philosophers’ stone of the meticulous human endeavor called “science,” is named after the Roman Goddess “Veritas” (Truth), the daughter of Cronus, the God of Time, and the mother of Virtus, the God of Values. This trilogy is illuminating. It shines light on the understanding that science is a child of its time and acknowledges that the search for Truth is always constrained by its historical era, colored by the beliefs and philosophies of its period, and limited by the tools available to its practitioners. But, moreover, it contends that scientific progress cannot be disconnected from the “life worth living” described by Aristotle. While originating in Time, science must breed “virtues,” else the search for truth may be meaningless. This lineage tells us that mythology viewed science as a concentrated, step-by-step effort that connects the past to the future, is responsive to the challenges of its time, and must be used in the service of society. It is of note that science is embodied by a woman - Veritas - the symbol of fertility and generation- to-generation continuity. With this in mind, let us contemplate the topic of “women in science.”

I begin with a disclaimer. In this brief opinion paper, I do not wish to provide a thorough discussion on the complex topic of science and women. It is a very broad topic often discussed from perspectives I have no knowledge of, such as post-positivistic, constructivist, and cultural feminism (e.g., Gergen, 2000; Hill et al., 2010). Nor do I touch upon the inequality in women being the subjects of science (Holdcroft, 2007). The goal here is to describe the personal road of a woman neuroscientist who has been doing research at the crossroad of psychology and neuroscience for three decades and to reflect on the difficulties, disappointments, misconceptions, prejudices, hopes, and achievements experienced along the way, alongside the gradual changes – in both research topics and opportunities - that have been taking place during this unique time-point in the history of women and science. My view is intimately tied to being a mother of four daughters and accompanying their struggle to carve a life that matters to them, both personally and professionally, in this generation of transition. It is also colored by the many female doctorate and post-doctorate students I mentored over the years and assisted their effort to become independent scientists, while, in most cases, also becoming mothers during the same intense period. This journey has convinced me that while the path to a position of authority in science is long and arduous for anyone, the road of a woman neuroscientist is far more precarious. It requires more perseverance, commitment, and outstanding brilliance, and, most importantly, desperately needs our support.

## Three perspectives on women in science

### Perspective one: veracity

As a first step to tackling our topic of women in science we must ask: what precisely is the “verifiable truth” of Edelman’s definition of science. A dictionary search for “veracity” reads as follows:

Veracity **noun**

ve·rac·i·ty və-ˈra-sə-tē

1. conformity with truth or fact: **ACCURACY**

2. devotion to the truth: **TRUTHFULNESS**

3. power of conveying or perceiving truth

4. something true (Merriam-Webster Dictionary, 2002).

As scientists, we can easily resonate with these descriptors of our efforts. Our daily work is a constant strive for accuracy, truthfulness, replicability, responsibility, undeniable evidence, and the power gained by passing the truth onward. However, if we apply this perspective to our topic, the issue of “women in science” becomes annulled.

The Veracity Perspective values outcome, not process, objectivity, not a subjective viewpoint. It considers truth to be a yes-or-no, right-or-wrong construct. This perspective rings the harsh tone of convictions, not the more tempered music that science is a child of its time and is fluid and changing. From this perspective, truth can be achieved by a man, woman, non-binary individual, or an alien from Mars. What matters for science is not who discovered it but that the truth has been revealed. Science is blind to the gender, race, age, culture, political camp, or religious affiliation of its practitioner. According to the Veracity Perspective, our topic “women in science” *is an oxymoron* (dictionary definition “a self-contradicting word or paradoxical group of words,” like “less is more”; Merriam-Webster Dictionary, 2002). “Women in science” is a non-issue, not because the problem has ceased to exist, but because it is inherently paradoxical; science, in essence, is genderless.

What can be wrong with such perspective? Well, plenty. And while this perspective has been adopted by many throughout the ages, it rests on a paradox. If science must utilize specialized tools, build on the knowledge of its time, become versed in past achievements, and scientists must spend time, concentrated effort, and long stretches of guided instruction to reach Truth, women, in the main, have been banned from such privileges, more fully in the past and more subtly nowadays. For centuries, women were not given the tools: education, time, status, freedom, financial support, mental encouragement, or early education, which are required for the making of a great scientist. They also rarely received the acknowledgement, respect, and recognition for work well done, were banned from the big funding needed to conquer new frontiers, and were made to remain at the lower levels of the academic ladder. While Truth may be gender-blind, in the soccer field of science gender is as bright as the shirt color of the competing teams, visible even from the last rows of the stadium.

Remember Shakespeare’s sister? The one so eloquently described by Woolf (1929) in *A room of your own* (1929). This girl was potentially as talented as her brother William but illiteracy, authoritarian family life, and days of preparing meals, herding sheep, or washing clothes at the river bank left no time for idleness. Lack of encouragement, opportunities, and horizon limited her dreams. Can you imagine a woman aboard the Beagle? Glued to the telescope to discover the shape of Earth? Lying under a tree to watch the apple falling? Contending that Truth is gender-blind is not only incorrect, it’s misleading. Such argument has been used by academic, religious, and political leaders to suggest that women possess inferior intelligence, their objectivity is tarred by their strong emotions, and their concentration is narrowed by a petty focus on appearances. The strong among them were cast as “witches” and the more reactive as “hysterics.” The proof of the Veracity Perspective utilizes precisely that circular argument; look at the history of (genderless) science and see that women have never made important headways. Thus, while Veritas is symbolized by a female Goddess, her sisters across the ages have generally been banned from the gates of Truth.

The problem with the Veracity Perspective, however, is not limited to its unfairness. It touches upon the fundamental issue of Truth itself and how one goes about achieving it. Since Kant, philosophers have become increasingly aware that Truth is not an objective “something” that can be grasped independent of what can be perceived by our senses and comprehended by our neural machinery. Twentieth century thought, particularly Husserl’s phenomenology and Wittgenstein’s philosophy of language have further emphasized the inter-dependence of the perceiver and the perceived. The Heisenberg principle has gone even further to argue for the inseparateness of the viewer and its subject at the level of “matter” itself and tells us that even the laws of nature are altered by the viewpoint of the observer. These notions challenge the very “objectivity” of science and indicate that even the most rigorous experiments are bound by the subjective perspective and specific tools of the practitioner. Whether or not women have a distinct viewpoint on evidence - do women tend to see matter more as “particles” than “waves” as compared to men – is a different question, but the topic of “women in science” cannot be described as an oxymoron if we take a look at the statistics. It is not intelligence women lack, as shown by extant research comparing the IQ of women and men (Halpern and Worell, 2001; Hunt, 2010), but a pathway.

### Perspective two: harsh reality

The Harsh Reality perspective on “women in science” builds bottom-up and looks at the numbers, and the numbers are indeed disheartening. Table 1 presents the number of Nobel Prize recipients by gender, representing the top human scientific achievements of the past 120 years. It is almost embarrassing to look at this figure. Women received but 6.7% of the Nobel prizes across the past century, and the proportions of women in the sciences: chemistry, physics, economics, and physiology or medicine, is even lower, standing at 3.6%. The fact that 96.4% of the Nobel Prizes in science were won by men makes it doubtfully clear that, as of today, women have not achieved the highest levels of science nor made the most impactful revelations.

**Table 1:** Nobel prize awards by gender in each category: 1901–2022.

Nobel prize	Female number	Male number
Chemistry	8	183
Economics	2	90
Literature	17	102
Peace	18	92
Physics	4	218
Physiology or medicine	12	213
Total	61	898

Still, while these numbers are disheartening, their interpretation is difficult. Extensive evidence suggests that women’s work and achievements are less credited and under- valued compared to that of men across all academic ranks (Ross et al., 2022; Ceci et al., 2023). Without a major social change we will not be able to tease apart the reasons why men have outperformed women in making the most notable scientific breakthroughs.

Let us look at another set of statistics that is not independent of the previous set. Data from Western countries, from which the vast majority of Nobel Prize winning scientific discoveries emanate, show a marked gender inequality at the top levels of academia.

In Europe:

- The Higher up the Academic Ladder, the Wider the Gender Gap: In 2018 Women accounted for 26.2% of Grade A positions, 41.8% of Grade B positions, and 46.9% of Grade C positions.
- Women are a minority among senior academics (Grade A) in many European countries, including the Netherlands (22.3%), Germany (20.5%), France (27.6%),
  Switzerland (24.1%), Sweden (28.2%) and the United Kingdom (26.4%) as of 2018 (European Institute for Gender Equality, 2019b).
- European women hold few positions in academic leadership. In the EU-28, women were only 23.7% of heads of higher education institutions in 2019 (European Institute for Gender Equality, 2019a).

In the UNITED STATES

- Women Are Less Likely to Achieve Tenure and Hold High Ranking Positions
- While women in the United States held nearly half (51.1%) of all tenure-track positions in 2021, they held just 40.6% of tenured positions.
- Women are more likely to be found in lower-ranking academic positions
- While women represent just over half (53.9%) of Assistant Professors and are near parity (47.3%) among Associate Professors, they accounted for barely over a third (35.8%) of Full Professors in 2021.
- Women held over half (57.2%) of all instructor positions, among the lowest ranking positions in academia.
- 22.5% of women faculty were in non-tenure-track positions, compared to 18.4% of men faculty in 2021 (National Center for Education and Statistics, 2021).
- Women of Color Are Especially Underrepresented in Academia as they held 10% of full-time faculty positions in 2019, a twofold underrepresentation compared to general US population (Colby and Fowler, 2020).

Female PhD recipients in all fields in the United States in 2020–2021 comprised 56%. Female PhD recipients in STEM field in the United States in 2020–2021 comprised 35% (National Center for Education and Statistics, 2022). Female PhD recipients in social sciences in the US in 2018–2019 comprised 47.1% (National Center for Education and Statistics, 2019).

What can we learn from these statistics? Several points come to mind that should guide us, women (and men) scientists of our generation, in our efforts to open new vistas for our daughters. First, women start in academia at basically the same place as men. For instance, data from the World Bank show that across the globe, in both high and middle income countries and across five continents, the proportion of female PhD recipients in natural sciences, mathematics, and statistics is approximately 50%. These fields were commonly perceived not only as closed to women but as those that require analytic thinking that does not fit the “female” form of more interpersonal and emotional IQ. The number of female PhD recipients in the social sciences is even higher. These numbers show that women can produce innovative contributions and reach initial excellence, the type of excellence that merits a doctorate degree, in these fields to the same extent as men (The World Bank, 2021). Still, when women finally make scientific discoveries, their research receives less publicity; it has been shown that papers whose first and last authors were female received significantly less citations as compared to papers in the same journals in which at least one of the lead authors was a man (Dworkin et al., 2020). This situation is not identical across all fields and the near absence of women at the top is particularly noted in STEM: Science (biological sciences, chemistry, physiology or medicine), Technology, Engineering, and Math (Hill et al., 2010; Ross et al., 2022).

With these numbers in mind, the main question we need to address is; what are the reasons that cause women to lag behind on the long road from earning a PhD to getting the rank of full professor, becoming a university president, or leading a well-funded lab? Why do women remain at the lower positions in academia, such as instructors or non-tenured lecturers, and do not reach the top. I believe there are two types of answers that need to be understood in order to find suitable solutions for the younger generation of female scientists.

The first set of reasons considers internal causes. It relates to issues such as motivation to achieve the highest levels of science that requires full undivided attention, long hours of work, gradual rise in income, stubbornness, and, to some extent, single- mindedness. Here we need to differentiate autonomous choices - women may not choose to have a singular focus, long hours, and, at some level or at least initially, exclusion of extensive social, cultural, and family activities. Being a top scientist necessitates significant personal compromises across many decades and it is possible that many women choose to carve a career that allows for more time to family, childrearing, friends, community, or the arts. And we still don’t have any data to show that reaching the highest levels in science is a better road to life’s happiness, fulfillment, or satisfaction.

But there are other internal reasons that must be addressed through education, role modeling, and social policy change. Often the life stories of women who reached high achievements – in science, literature, politics, or the arts – included someone who believed in them early on, saw their special talent, and pushed them to achieve, whether a parent, teacher, or mentor with whom the young girl had an affiliative bond. Girls who are not encouraged may not believe they can aim high in science, and, as a society, we should make sure that those who show talent, curiosity, and inclination can have the encouragement they need and a variety of early scientific experiences for their participation.

Similarly, women are still the ones responsible for childcare, those who take time off after the birth of a baby, and those responsible for most of the housekeeping and childcare chores and the years of childbearing are typically those in which individuals make their greatest investment in a scientific career, particularly a career that aims high. Again, it’s very difficult to disentangle women who take time off because this is their inner wish from those who take maternity leave because state policies do not afford a paternal leave, those who have no circle of support, or those who do it because society has placed this role on their shoulders.

The second set of reasons relates to external causes and these should be changed through policy and targeted effort to make sure that talented young girls and women who choose to devote their life to science can actualize their dream. Education, for instance, from a young age, that involves encouragement, opportunities already set in elementary school, mentorship by senior female scientists at a stage when identity is formed, and culture- specific and race-specific role models. For instance, girls from a minority background should be exposed to top female scientists from marginalized or minority groups countries. We should offer special tutoring to help girls believe in themselves and models to show that the road to excellence is possible to all. Social policies that involve time off for fathers after childbirth, education for father involvement, and a corporate atmosphere that accepts and even encourages fathers to take time for active parental care are very much in need.

While gradual changes in family role division and father involvement have been taking place over the last three decades, women are still responsible for the vast majority of housekeeping and childcare activities, even in dual-earner egalitarian families. Much further change is needed to enable women the peaceful alone states that are required for Archimedes’ legendary “Eureka.”

Finally, some tough questions need to be addressed with regards to the Harsh Reality perspective. I remember the first weeks of my post-doctorate at the Yale Child Study Center. The legendary director, Dr. Donald Cohen, met with each of us young post-docs who were carefully selected from across the world for the prestigious position. Looking at my CV, he said something I never forgot: “in the top left hand of your CV you should put “married plus four children (the fifth was born at the end of my post-doc), but the rest of your CV should look like that of a single male.” Should it? I wonder. And this is a truly tough question. On the one hand, we do not want women to reach top scientific positions or receive lucrative scientific prizes by “quota.” Each of us wants to know that we got to where we got by merit, not by mercy. On the other hand, are there not concessions we need to make for the fact that women carry the burden of pregnancy and childbirth, that the work–family conflict is much tougher for women, and that most of us do not have the “wife” needed for the making of the great scientist? There are also historical wrongs that need some righting and the “all boys network,” so common in politics and the corporate world, is still very much alive in science and should be abolished. I do not have the answer but assume that, like much else in life, the answer is less of a “yes-or-no” and more of a blend that acknowledges the complexity.

### Perspective three: advantage?

Unlike the first two perspectives, I put this perspective with a question mark and approach it with extreme caution. Can being a women offer some advantage in the acquisition of cumulative knowledge? The caution here is due precisely to the opinions highlighted in the *Veracity Perspective*, those attitudes that have been abused by men of power since antiquity and have recently become more common in various spots across the world. These opinions contend that women have a unique type of intelligence that is emotional and relational, that they are biologically-prepared for raising children, and that their “natural” place as nurturers is the kitchen. Suggesting that women, as a group, may do science differently is shooting us in the foot.

Still, I would like to utilize perspectives that matured throughout the 20th century and my research in *affiliative neuroscience* to highlight differences between women and men in the pathways to the parental brain and from these findings suggest some possible uniqueness in a woman’s perspective. These suggestions must be constrained by the understanding that the “maternal” and “paternal” pathways to the parental brain are two potential pathways that exist in both women and men and individual differences define the degree of their expression in each individual. This is beautifully exemplified by our work on the parental brain in gay fathers who are raising infants born by surrogacy without maternal involvement.

One of the important progressions, in my opinion, in 20th century thought involved postulating philosophical models that turned away from the metaphysical and abstract to focus the lens on the contextual and daily as a potential road to Truth. Such models include Husserl’s (1977) phenomenology, which argues for the “aboutness” of knowledge, Merleau-ponty’s (1945) emphasis on lived experiences, and Santayana’s (1904) writing on perceptual “essences” as the foundations for mental life. Models in cognitive neuroscience adapted the perspective of Varela et al. (2016) on cognition as geared to solve daily problems and discussed how cognitive faculties are molded by the specific problems individuals encounter in their physical and social ecology. These authors grounded mental life in the contextual and local, refuted schools of thoughts that emphasized abstract or metaphysical “ideas” (e.g., Plato, Descartes), and argued that human knowledge is eclipsed by the human body and its affordances and limitations. This ushered current perspectives on “interoception,” attention to the bodily milieu, and its role in mental health and development. The work of these philosophers may suggest that women and men, or at least in their roles as mothers and fathers, may activate different sets of behaviors, potentiate somewhat specific neural pathways, and trigger unique physiological support systems that sustain their parental role and these may contribute in distinct ways to children’s ability to navigate their social world.

Our model on the “neurobiology of love (and hatred)” (Feldman, 2020a,b, 2021, 2023) posits three foundations that underpin the human capacity for affiliation, love, and attachment. These foundations are first cemented in the infant’s relationship with mother and father, and then transfer to other affiliative bonds the child forms throughout life; with close friends, romantic partners, mentors, or therapists, and eventually with his or her own children. The first foundation is the process of *biobehavioral synchrony*, the creation of a coupled biology between affiliative partners through the coordination of nonverbal social cues (Feldman, 2007, 2012, 2016). The second foundation is the *oxytocin* system in its role as an integrative interface that connects the reward, stress, and immune systems in the formation of affiliative bonds and the maturation of social skills that enable children to participate in human societies. The third foundation is the *affiliative brain*, the neural network that integrates the subcortical structures sustaining mammalian maternal care with cortical networks implicated in embodies simulation, mentalization, and emotion regulation. These bottom-up and top-down structures cohere into an “attachment network” that is triggered in each individual by the early care received from parents and then functions to sustain human love throughout life, as seen in studies of parental, romantic, and close friendship relationship (Feldman, 2020a,b Ulmer-Yaniv et al., 2022).

Each of these three foundations is formed in a unique way in mothers and fathers and expresses distinctly in the infant’s relationship with each parent. Synchrony with mother and father is express in different types of rhythms; we call them “the rhythm of safety” versus “the rhythm of exploration” (Feldman, 2003). Synchrony with mother cycles between states of medium and low arousal and centers around face-to-face communication, gaze synchrony, and the matching of vocalizations, affect, and touch. It directs infants inward, to the interacting dyad, and trains them to pay close attention to the moods, mental states, intentions, and desires of their social partners without the need for words. Father-infant synchrony, which is just as tight, turns outward toward the environment and its wonders, focuses on joint exploration of objects and exciting events, and contains quick and random peaks of positive arousal. In health, when the infant’s relationship with the two parents is synchronous, infants internalize that close relationships contain components of security, predictability, and attunement to ongoing facial signals, as well as excitement, exploration, and adventure in the outside world, and that he or she can find security and excitement in the context of close relationships (Feldman, 2023).

Oxytocin, implicated in birth and lactation, shows similar levels in new mothers and fathers when not tested immediately after breastfeeding. However, oxytocin levels in mothers are linked with the maternal relational style of shared gaze, “motherese” vocalizations, and affectionate touch, while levels in fathers are related to high positive arousal, object focus, and stimulatory contact (Gordon et al., 2010). This indicates that the biological foundations of bonding are tuned by mother and father to different potentials and these distinct potentials may be expressed within the child’s future close relationships. In health, when both mother and father formed a secure relationship with the child, he or she can find in close attachments security and excitement, interpersonal focus and exploration, attention to non-verbal social cues with joint adventure in the outside world.

Finally, with regards to the “parental brain,” the neural network that sustains attachment, the maternal pathway to consolidation of the “parental brain” is more evolutionarily ancient, subcortical, and bottom up, is triggered by the high oxytocin rush during labor, is linked with the maternal form of interpersonal synchrony (Shimon-Raz et al., 2021). In contrast, the paternal pathway to the “parental brain” activates networks involved in mentalization and cognitive control, recruits top-down processing, and is triggered by the father’s involvement with daily infant care and the constant reading of the child’s mental states and social signals. Importantly, however, fathers can activate the automatic, bottom-up network by assuming responsibility and investing in the caregiving of infants. This is evident, for instance, in gay fathers who are raising infants without maternal care, but also appears in heterosexual fathers who spend time alone with the infant and assume the full range of parental responsibility, from feeding and bathing to doctor’s visits and cognitive stimulation (Abraham and Feldman, 2022). These findings highlight the immense neural plasticity that accompanies attachment behavior and advocate that alterations in policies, habits, norms, and practices can bring about neural changes in the brain basis of attachment and love.

While in no way can we infer from these findings that women’s road to the “verifiable truth” differs from that of men – and the past shows us that when it comes to gender, race, or minority status “different” always opens the door to “inferior” – isn’t is likely that, least in some domains, women may have some insights that open new vistas to knowledge? Women’s life experiences may also cultivate special sensitivities that can offer a fresh outlook and new directions for research. The recent focus on interactive synchrony, cross- brain communication, and the oxytocin system and the emergence of new fields in neuroscience, such as social and affiliative neuroscience, clearly tell us that topics that have been considered unimportant and marginal only a decade ago have now moved to the foreground of neuroscience and are researched by the best minds of both female and male scientists. In light of studies indicating that being a top scientist does not necessarily go with high level of well-being (Pace et al., 2021), women may chart a new model for a successful scientific career that is not built on a total solitary focus but on a more balanced path that could better fit the life circumstances of women and minorities. Our special time in history offers unique opportunities to women in the sciences. We should make sure not to miss this moment so that the road of our daughters may be easier than our own and they can fulfil the entire range of possibilities their dreams may take them.

Tough topics, particularly during moments of historical transitions, always invite more questions than answers. Knowing that the road ahead is long and arduous, let us conclude with the touching words of W. Dabney Stuart, a right-on-target poem that links women and science.

“That miracle of

Science the Half

Man Half Woman

Divided into a garden

Of Eden where only the one who

Offers the apple eats”

While women throughout the ages have been limited to “offering the apple,” it is time we learn to both share the apple and eat it.

## Data availability statement

The original contributions presented in the study are included in the article/supplementary material, further inquiries can be directed to the corresponding author.

## Author contributions

RF conceived and wrote the paper.

## References

[ref1] Abraham E. Feldman R. (2022). The neural basis of human fatherhood: a unique biocultural perspective on plasticity of brain and behavior. Clin. Child. Fam. Psychol. Rev. 25, 93–109. doi: 10.1007/s10567-022-00381-9, PMID: 35122559

[ref2] Ceci S. J. Kahn S. Williams W. M. (2023). Exploring gender bias in six key domains of academic science: an adversarial collaboration. Psychol. Sci. Public Interest 24, 15–73. doi: 10.1177/15291006231163179 37098793 PMC10394402

[ref3] Colby G. Fowler C. (2020). Data snapshot: IPEDS data on full-time women faculty and faculty of color. Available at: https://www.aaup.org/sites/default/files/Dec-2020_Data_Snapshot_Women_and_Faculty_of_Color.pdf

[ref4] Dworkin J. D. Linn K. A. Teich E. G. Zurn P. Shinohara R. T. Bassett D. S. (2020). The extent and drivers of gender imbalance in neuroscience reference lists. Nat. Neurosci. 23, 918–926. doi: 10.1038/s41593-020-0658-y, PMID: 32561883

[ref5] Edelman G. M. (1972). Banquet speech at the Nobel banquet in Stockholm The Nobel Prize Available at: https://www.nobelprize.org/prizes/medicine/1972/edelman/speech/.

[ref6] European Institute for Gender Equality. (2019a). Heads of institutions in the higher education sector (HES): Number and distribution (%) by sex. Available at: https://eige.europa.eu/gender-statistics/dgs (Accessed September 12, 2023).

[ref7] European Institute for Gender Equality. (2019b). Proportion (%) of women among academic staff/researchers in the higher education sector, by grade and in total.

[ref8] Feldman R. (2003). Infant-mother and infant-father synchrony: the coregulation of positive arousal. Infant Ment. Health J. 24, 1–23. doi: 10.1002/imhj.10041

[ref9] Feldman R. (2007). Parent–infant synchrony: biological foundations and developmental outcomes. Curr. Dir. Psychol. Sci. 16, 340–345. doi: 10.1111/j.1467-8721.2007.00532.x

[ref10] Feldman R. (2012). Oxytocin and social affiliation in humans. Horm. Behav. 61, 380–391. doi: 10.1016/j.yhbeh.2012.01.008 22285934

[ref11] Feldman R. (2016). The neurobiology of mammalian parenting and the biosocial context of human caregiving. Horm. Behav. 77, 3–17. doi: 10.1016/j.yhbeh.2015.10.001 26453928

[ref12] Feldman R. (2020a). The biology of love AEON Available at: https://aeon.co/essays/why-care-and-the-scare-are-inseparable-when-you-love-someone.

[ref13] Feldman R. (2020b). What is resilience: an affiliative neuroscience approach. World Psychiatry 19, 132–150. doi: 10.1002/wps.20729 32394561 PMC7215067

[ref14] Feldman R. (2021). “The neurobiology of affiliation; maternal-infant bonding to life within social groups” in Encyclopedia of behavioral neuroscience. 2nd *Edn*. Ed. Sala S. D. (Amsterdam: Elsevier), 518–531.

[ref15] Feldman R. (2023). The neurobiology of hatred: Tools of Dialogue© intervention for youth reared amidst intractable conflict impacts brain, behaviour, and peacebuilding attitudes. Acta Paediatr. 112, 603–616. doi: 10.1111/apa.16676, PMID: 36655828

[ref16] Gergen M. (2000). Feminist reconstructions in psychology: narrative, gender, and performance. Thousand Oaks: Sage Publications.

[ref17] Gordon I. Zagoory-Sharon O. Leckman J. F. Feldman R. (2010). Oxytocin and the development of parenting in humans. Biol. Psychiatry 68, 377–382. doi: 10.1016/j.biopsych.2010.02.005 20359699 PMC3943240

[ref18] Halpern D. F. Worell J. (2001). “Sex difference research: cognitive abilities” in Encyclopedia of women and gender: sex similarities and differences and the impact of society on gender. ed. Worrel J., vol. 2 (San Diego: Academic Press), 963–971.

[ref19] Hill C. Corbett C. St Rose A. (2010). Why so few? Women in science, technology, engineering, and mathematics. Washington, DC: Association of University Women.

[ref20] Holdcroft A. (2007). Gender bias in research: how does it affect evidence based medicine? J. R. Soc. Med. 100, 2–3. doi: 10.1258/jrsm.100.1.2 PMC1761670 17197669

[ref21] Hunt E. (2010). Human intelligence. Cambridge Cambridge University Press.

[ref22] Husserl E. (1977). Phenomenological psychology (J. Scanlon, trans.) The Hague: Martinus Nijhoff.

[ref23] Merleau-ponty M. (1945). Phénoménologie de la perception (1945) Motivation. Paris, France: Reprint Paris Gallimard.

[ref24] Merriam-Webster Dictionary. (2002). Merriam-Webster dictionary. Springfield Merriam-Webster, Inc.

[ref25] National Center for Education and Statistics. (2019). Degrees in the social sciences and history conferred by postsecondary institutions, by level of degree and sex of student: 1970–71 through 2017–18. Available at: https://nces.ed.gov/programs/digest/d19/tables/dt19_325.90.asp

[ref26] National Center for Education and Statistics. (2021). Number of full-time instructional staff by gender, faculty and tenure status. Available at: https://nces.ed.gov/ipeds/TrendGenerator/app/build-table/5/51?rid=163&cid=165

[ref27] National Center for Education and Statistics. (2022). Number and percentage distribution of science, technology, engineering, and mathematics (STEM) degrees/certificates conferred by postsecondary institutions, by race/ ethnicity, level of degree/certificate, and sex of student: academic years 2011–12 through 2020–21. Available at: https://nces.ed.gov/ipeds/search?query=phd%20stem%20gender&query2=phd%20stem%20gender&resultType=all&page=1&sortBy=date_desc&overlayDigestTableId=202325

[ref28] Pace F. D’Urso G. Zappulla C. Pace U. (2021). The relation between workload and personal well-being among university professors. Curr. Psychol. 40, 3417–3424. doi: 10.1007/s12144-019-00294-x

[ref29] Popper K. R. (1959). The logic of scientific discovery. London: Routledge.

[ref30] Ross M. B. Glennon B. M. Murciano-Goroff R. Berkes E. G. Weinberg B. A. Lane J. I. (2022). Women are credited less in science than men. Nature 608, 135–145. doi: 10.1038/s41586-022-04966-w, PMID: 35732238 PMC9352587

[ref31] Santayana G. (1904). What is aesthetics? Philos. Rev. 13:320. doi: 10.2307/2176284

[ref32] Shimon-Raz O. Salomon R. Bloch M. Romano G. A. Yeshurun Y. Ulmer-Yaniv A.. (2021). Mother brain is wired for social moments. elife 10, 1–32. doi: 10.7554/eLife.59436 PMC8026217 33764299

[ref33] The World Bank (2021). Share of graduates by gender, female (%). Washington, DC: The World Bank’s Gender Data Portal.

[ref1001] Ulmer-Yaniv A. Waidergoren S. Shaked A. Salomon R. Feldman R. (2022). Neural representation of the parent-child attachment from infancy to adulthood. Soci. Cogn. Affec. Neuroscie. 17, 609–624. 10.1093/scan/nsab132 PMC9250301 34893911

[ref34] Varela F. J. Thompson E. Rosch E. Kabat-Zinn J. (2016). “The embodied mind: cognitive science and human experience” in The embodied mind: cognitive science and human experience (Cambridge, MA: The MIT Press).

[ref35] Woolf V. (1929). A room of one’s own. London, England: Routledge.

